# 

**Published:** 1920-06-05

**Authors:** 


### The College of Nursing (Limited by Guarantee).

HOURS OF EMPLOYMENT BILL.

The following scheme is suggested by the College of Nursing to be included in a Special Order in the Hours of Employment Bill:

1. That all registered nurses and other persons actually engaged in rendering services in direct connection with the nursing of the sick, excepting maternity nurses, be included in the provision of the Special Order.

2. That for nurses in institutions for the sick, including those where probationers are in training, for nurses in district nursing institutions, and those employed by district nursing associations, and for nurses engaged in public-health work, the maximum working hours be fifty-six per week, taken over a period of four weeks, the time on duty not to exceed ten hours in twenty-four hours. Any time worked in excess of the maximum to be compensated by extra off-duty, given during the nurse's normal working hours, and if extra off-duty time due to the nurse exceeds forty-eight hours, board and residence to be provided, or the monetary equivalent, if preferred by the employer.

3. That in institutions for trained nurses on the co-operative system, and other institutions supplying nurses for private cases and in nursing homes, the maximum working hours be fifty-six per week, taken over a period of fourteen days, any time worked in excess of this maximum to be compensated by the same number of hours given as extra off-duty during the nurse's normal working hours, and the nurses to receive, in addition, the fee due from the patient for the extra time worked by her, together with board and residence, or the monetary equivalent, if preferred by the employer.

### Matrons' and Sisters' Appointments.

Hackney Union Infirmary.—Miss Edith Janetta Anderson has been appointed night superintendent nurse. She was trained at Bagthorpe Infirmary, Nottingham, where she was later ward sister, and she has since been health visitor and inspector of medicines at St. Helens. Miss Anderson holds the certificate of the Central Midwives Board.

Holme Valley Memorial Hospital, near Huddersfield.—Miss J. Dunning has been appointed matron. She was trained at the Royal Infirmary, Chester; has been sister at the Infirmary, Bury, the Royal Infirmary, Manchester; matron of the Cottage Hospital, Maryport, Cumberland; and sister-in-charge of the Military Hospital, Seymour Park, Old Trafford, Manchester.

Lowestoft and North Suffolk Hospital.—Miss C. Alhum, Miss E. Raven, and Miss I. M. E. Parkerson have been appointed sister of the women and children's wards, sister of the theatre and male wards, and night sister respectively. Miss Alhum was trained at King's College Hospital, and has been night sister and sister of the medical flat, Evelina Hospital for Sick Children. Miss Raven was trained at the Sheffield Royal Hospital, where she was later sister of the women's surgical wards. She has also been sister at the Ipswich Surgical Home and at the Birmingham General Hospital, and has served overseas with Queen Alexandra's Imperial Military Nursing Service Reserve. Miss Parkerson was trained at the Royal Halifax Infirmary, and has served overseas with the Territorial Force Nursing Service.

National Hospital for Diseases of the Heart, Westmoreland Street.—Miss Gertrude Morris has been appointed matron. She was trained at St. Marylebone Infirmary, where she was later ward sister, and she has been sister housekeeper and assistant matron at St. Pancras Infirmary, and sister at the 3rd London General Hospital.

North-Eastern Fever Hospital, South Tottenham.—Miss Lillian Gladys Burbidge has been appointed sister. She was trained at the Stanley Hospital, Liverpool, and at the Isolation Hospital, Wellington, New Zealand. She has held posts at the South Devon and East Cornwall Hospital, Plymouth; the Jenny Lind Hospital, Norwich; the Astley Sanatorium, near Manchester; and temporarily at the London Fever Hospital, Islington. Miss Burbidge served in Queen Alexandra's Imperial Military Nursing Service Reserve in Malta and Salonika. She is a member of the College of Nursing.

St. Marylebone Infirmary, Ladbroke Grove, W.—Miss M. O. Hayden and Miss Catherine McLennan have been appointed sister and ward sister respectively. Miss Hayden was trained at the Princess Christian Hospital, Weymouth, where she was later sister, and she has been sister at the St. John Ambulance Brigade Hospital, Etaples and Trouville, and also in Syria; she has also done private nursing. Miss McLennan was trained at the St. Marylebone Infirmary, and was later staff nurse there.

### Recent Official Publications.

(To be obtained from H.M. Stationery Office, Imperial House, Kingsway, W.C. 2 Prices include postage.)

Dysentery and Certain Other Tropical Diseases; Trench Fever; Gas-Poisoning and its Sequelæ in Relation to Disabled Ex-Service Officers and Men. Notes and Suggestions. 1920. 7½d.

Overseas Settlement. Professional Handbook, Part II. Chemists and Druggists, Nurses, Physicians and Surgeons, Veterinary Surgeons. 7½d.

Bill. Dangerous Drugs. To regulate the Importation, Exportation, Manufacture, Sale and Use of Opium and other Dangerous Drugs. 3d.

Public Opinion on Preventive Medicine. The Lady Priestley Memorial Lecture, by Sir George Newman, K.C.B., M.D., F.R.C.P., Chief Medical Officer, Ministry of Health. 5½d.

